# Influence of Language Barriers on Postoperative Recovery After Hepatobiliary and Pancreatic Surgery: A Retrospective Analysis

**DOI:** 10.1002/wjs.70263

**Published:** 2026-03-02

**Authors:** Freya Brodersen, Jana Hinz, Sinja Friedl, Faik Güntac Uzunoglu, Asmus Heumann, Tarik Ghadban, Ramez Wahib, Thilo Welsch, Thilo Hackert, Sidra Khan‐Gökkaya

**Affiliations:** ^1^ Nursing Department of General‐, Visceral‐, and Thoracic Surgery University Medical Center Hamburg‐Eppendorf Hamburg Germany; ^2^ Nursing Department Center for Surgical Medicine University Medical Center Hamburg‐Eppendorf Hamburg Germany; ^3^ Department of General‐, Visceral‐, and Thoracic Surgery University Medical Center Hamburg‐Eppendorf Hamburg Germany; ^4^ Department of General, Visceral and Tumor Surgery Krankenhaus Nordwest Frankfurt Germany; ^5^ University Medical Center Hamburg Eppendorf Hamburg Germany

**Keywords:** ERAS, language barrier, perioperative care, surgery

## Abstract

**Background:**

Understanding health related information is crucial for informed consent and active participation in surgical care. Language barriers between patients and health care professionals pose the risk of misunderstandings and incomplete information exchange. This can significantly impair the quality of health care. Little is known about the impact of language barriers in hepatobiliary and pancreatic surgery and its consequences for postoperative recovery.

**Method:**

We performed a retrospective study in a University Medical Center in Germany assessing patients from 2020 to 2023 who underwent hepatobiliary or pancreatic surgery. Primarily we investigated whether length of stay (LOS) differed between patients with and without language barrier. Secondary we examined (Enhanced Recovery After Surgery) ERAS‐compliance, preoperative education, postoperative mobilization habits, readmission, mortality, occurrence of complications, and certain postoperative complications between both groups.

**Results:**

We included 848 patients in our study, 57 (6.5%) patients of whom had a language barrier. The length of stay did not differ significantly between the two groups (12.8 days; (CI 95%: 11.9–13.7) versus 14.4 days; (CI 95%: 11.1–17.7) (*p* = 0.320). The interpreting service in our cohort was rarely used overall. Patients with language barriers were younger (CI 95%: 46.7–56.7 vs. 59.9–61.9; *p* = 0.001) and differed in terms of their diagnoses (*p* = 0.001). We found no differences in ERAS Compliance, complication rate and mortality. Among secondary outcomes, patients with language barriers showed higher rates of specific postoperative complications, including pulmonary embolism (*p* = 0.026), and paralytic ileus (*p* = 0.047). Patients without language barriers were more likely to be mobilized on day of surgery (*p* = 0.009) and received preoperative ERAS‐education more frequently (*p* = 0.035).

**Conclusion:**

Patients experiencing language barriers constitute a small group. Length of stay did not differ between the two groups. However, with respect to postoperative complications further investigation with larger patient cohorts is needed. Our findings emphasize the need for additional research and development of practical and patient‐centered strategies to effectively address language barriers in clinical care.

## Graphical abstract

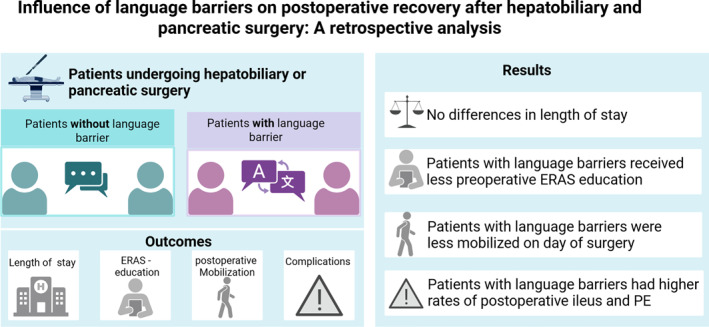

## 1 Background

Language plays a crucial role in effective communication, particularly in health care settings [1]. When patients and health care providers do not speak the same language, a language barrier is present. There is evidence that language barriers can severely affect the quality of health care [2, 3, 4, 5, 6, 7]. For example, language barriers make it difficult to obtain medical histories, impede informed consent, lead to misunderstandings, endanger patient safety, and ultimately affect patients' health outcomes [2, 3, 4, 5, 6, 7, 8, 9]. This could be demonstrated especially for chronic conditions [4, 6]. Patients with language barriers have poorer access to common health services and fewer medical consultations and preventive check‐ups [2, 10, 11, 12, 13]. Especially in oncological treatment, the consequences are severe. Patients receive later access to oncological treatment and present themselves at a more advanced cancer stage at the beginning of therapy [14, 15]. In pancreatic cancer, language barriers are associated with worse survival [14]. Moreover, language barriers increase the workload of health care professionals and lead to greater costs for the health care system [16, 17, 18, 19]. In addition, patients with language barriers seem to be underrepresented in clinical research because language barriers are often an exclusion criterion for clinical trials [20]. To make matters worse, many language barriers are not even recognized in clinical practice [2, 21].

Professional interpreters can bridge barriers but are not frequently used [15, 21, 22]. Thus, affected patients and health care providers often must interact with medical unexperienced bilingual relatives, translation apps, or nonmedical bilingual professionals [21].

Surgery is an important treatment for various conditions and requires patients to be well informed and to actively participate for enhanced recovery. The impact of language barriers on postoperative recovery has been poorly studied. Nevertheless, recent findings are equally concerning. A few studies have shown that patients with language barriers who undergo surgery have an increased length of stay (LOS), higher rates of readmission and more surgical site infections [18, 19, 23, 24, 25, 26, 27, 28]. Postoperative pain management seems to differ between patients with and without language barriers. Plancarte et al. reported that patients with language barriers received medication later and in lesser amounts than did patients without language barriers [29]. In terms of postoperative mortality, studies have shown ambiguous results [7, 24, 28, 30, 31]. Castro et al. reported higher mortality rates among patients with language barriers in the acute trauma setting [28]. However, robust data and detailed studies in the field of surgery are still rare, especially in Germany [23].

Major visceral surgery, such as pancreatic and hepatobiliary surgery, is associated with frequent complications and relatively high mortality rates [32, 33, 34, 35]. Enhanced Recovery after Surgery (ERAS) pathways can improve recovery after major surgery by reducing the LOS and incidence of postoperative complications [36, 37]. ERAS is an evidence‐based multimodal treatment pathway that contains elements in which patients are actively involved and elements in which health care providers play an executive role. Elements such as early mobilization, carbohydrate loading and early oral intake of food are components for which collaboration from patients is needed. Omitting drains, multimodal analgesia and early catheter removal are components of the ERAS pathway in which health care providers play an executive role [38]. However, little is known about the impact of this pathway in patients with language barriers. Since education and participation in treatment are crucial components of ERAS, we hypothesized that language barriers might have a severe impact on postoperative recovery. The purpose of this study was primarily to examine the impact of language barriers on surgical outcomes and recovery after hepatobiliary and pancreatic surgery within an ERAS setting.

## 2 Methods

We performed a retrospective observational study at an ERAS‐certified university medical center in Germany. The conception and reporting of this study were designed according to the Strengthening the Reporting of Observational Studies in Epidemiology (STROBE) guidelines [39]. All patients who underwent elective pancreatic or hepatobiliary surgery were included.

A language barrier was defined by the necessity of an interpreting service for surgical consent prior to surgery. Interpreting service involvement during the hospital care is automatically recorded in the electronic patient record as a consultation event, which enables also the identification of the frequency of interpreter use and the patient's language. We compared the outcomes of patients with language barriers to those of patients without language barriers. Patients agreed to participate in prospective data collection and evaluation as part of our ERAS program, an approval from an ethics committee is in place. We extracted data from January 2020 to March 2023 from medical records and the ERAS Interactive Audit System (EIAS). The EIAS is a prospective database for the auditing and evaluation of ERAS implementation [40]. The database creates a value that reflects adherence to the corresponding ERAS guidelines, called ERAS compliance. The patients were routinely contacted 30 days after surgery to assess complications and readmission after discharge.

The primary outcome in our study was the length of hospital stay (LOS). As secondary outcomes, we evaluated ERAS compliance, complication, readmission and in‐hospital mortality rates. To report the occurrence of specific complications, we decided to focus on complications in which patient engagement could play a role. Accordingly, we recorded the occurrence of the following complications: pneumonia, pleural effusion, respiratory failure, wound infection, urinary tract infection, deep venous thrombosis, pulmonary embolism, acute kidney injury and paralytic ileus. We also evaluated whether patients usually received preoperative ERAS education from an ERAS nurse and postoperative mobilization as confounding variables. Postoperative mobilization was defined as a dichotomous outcome requiring mobilization to at least a sitting or standing position.

Multivisceral resection was defined as additional resection of one or more adjacent or distant organs or partial resection of organs that are not usually resected during pancreatic or hepatobiliary surgery [41, 42]. Splenectomy associated with distal or total pancreatic resection was not considered multivisceral resection. Appendectomy and cholecystectomy were also not considered major resection and were therefore not included in this definition.

We used SPSS (IBM SPSS Statistics 29.0.1.0) for data analysis. For the comparison of parametric data, the *t* test for independent samples was used. Differences in results were considered statistically significant if the *p* value was < 0.05. We analyzed nominal data with the Chi^2^ test and used the Mann‒Whitney *U* test to compare nonnormally distributed parametric data. Incomplete patients' datasets were carefully explored. If only certain variables were missing, the patients were included in the analysis. Patients whose data were predominantly incomplete were excluded. We accounted for influence of potential confounding factors using multiple linear regression for length of stay. After the initial data review, we determined that the two groups were not fully comparable. We therefore followed the data by performing an additional subgroup analysis including patients with oncological diseases.

## 3 Results

We included a total of 848 patients in our study. Fifty‐seven patients (6.5%) experienced language barriers and received professional interpretation services during primary surgical consultation. A total of 37.5% of the patients with language barriers had only one contact with a professional interpreter during preparation for surgery and the whole hospital stay. The average number of contacts with a professional interpreter per person was 1.6 consultations during the entire treatment process in our department (ambulatory preparation for surgery and primary length of stay). Among patients with language barriers, a total of 19 different languages were spoken. The most represented languages were Turkish (15.5%) and Persian (15.8%), followed by Russian (10.5%), Bulgarian (7.0%) and Kurdish (7.0%). The majority of patients in both groups were male, with slightly more male patients in the language barrier group (Table 1). On average, patients with language barriers were almost 10 years younger than patients without language barriers (60.9 ± 14.3 vs. 51.7 ± 18.9 years; *p* = 0.001). Surgical procedures, American Society of Anesthesiologists (ASA) classification did not differ significantly between the groups, and the frequency of multivisceral resection was similar. Both groups showed no significant differences with respect to comorbidities, smoking status, or excessive alcohol consumption (Table 1). We observed significantly different distributions of final diagnoses between the two groups. Notably, almost a quarter of patients with language barriers were receiving treatment for echinococcosis, whereas fewer than 1% of patients without language barriers were receiving treatment for echinococcosis. Furthermore, patients with echinococcosis were much younger than other patients who underwent surgery for malignant diseases (mean 35.9 vs. 60.4 years). In addition, patients with language barriers rarely underwent surgery due to other benign diseases or disorders (excluding chronic pancreatitis) compared with patients without language barriers.

**TABLE 1:** Patient characteristics (overall study cohort).

Variable	No language barrier (*n* = 791)	Language barrier (*n* = 57)	*p* value
Anthropometrics			
Female *n* (%)	367 (46.4)	19 (33.3)	0.056
Age [avg/SD]	60.9 ± 14.3	51.7 ± 18.9	**0.001**
BMI [avg/SD]	25.3 ± 5.1	25.8 ± 5.9	0.409
ASA classification *n* (%)			0.260
I	21 (2.7)	4 (7.0)	
II	312 (39.4)	23 (40.4)	
III	441 (55.8)	28 (49.1)	
IV	13 (1.6)	1 (1.8)	
Missing	4 (0.5)	1 (1.8)	
Consumption of noxious substances *n* (%)			
Alcohol	74 (9.4)	3 (5.3)	0.689
Missing	13 (1.6)	1 (1.8)	
Smoking	180 (22.8)	15 (26.3)	0.863
Missing	12 (1.5)	1 (1.8)	
Comorbidities *n* (%)			
Diabetes mellitus	150 (19.0)	13 (22.8)	0.658
Asthma	39 (4.9)	4 (7.0)	0.661
COPD	30 (3.8)	1 (1.8)	0.605
Cardiovascular disease	377 (47.7)	21 (36.8)	0.221
Previous thromboembolic event	76 (9.6)	4 (7.0)	0.670
Chronic kidney disease	39 (4.9)	2 (3.5)	0.738
Steatotic liver disease	43 (5.4)	4 (7.0)	0.739
Chronic hepatitis	51 (6.4)	8 (14.0)	0.081
Livercirrhosis	53 (6.7)	3 (5.3)	0.759
Surgical procedure *n* (%)			0.087
PD	72 (9.1)	2 (3.5)	
PPPD	72 (9.1)	4 (7.0)	
DPPHR	63 (8.0)	5 (8.8)	
Distal resection	67 (8.5)	3 (5.3)	
Total pancreatectomy	51 (6.4)	2 (3.5)	
Other pancreatic surgery	28 (3.5)	2 (3.5)	
Right hemihepatectomy	25 (3.2)	6 (10.5)	
Extended right hemihepatectomy	30 (3.8)	6 (10.5)	
Left hemihepatectomy	24 (3.0)	3 (5.3)	
Extended left hemihepatectomy	14 (1.8)	1 (1.8)	
Wedge oder minor resection	155 (19.6)	11 (19.3)	
Other segmentectomies	152 (19.2)	11 (19.3)	
Other biliary surgery	38 (4.8)	1 (1.8)	
Surgical approach *n* (%)			0.07
Open	558 (70.5)	40 (70.2)	
Laparoscopic	94 (11.9)	6 (10.5)	
Robotic	112 (14.2)	5 (8.8)	
Converted to open	26 (3.3)	6 (10.5)	
Multivisceral resection *n* (%)	145 (18.3)	8 (14.0)	0.415
Final diagnosis *n* (%)			**0.001**
Pancreatic cancer	210 (26.5)	10 (17.5)	
NET	18 (2.3)	2 (3.5)	
Chronic pancreatitis	74 (9.4)	4 (7.0)	
Benign pancreatic cyst	9 (1.1)	2 (3.5)	
IPMN/PanIN	16 (2.0)		
Hilar cholangiocarcinoma	43 (5.4)	4 (7.0)	
Intrahepatic cholangiocarcinoma	37 (4.7)	2 (3.5)	
HCC	69 (8.7)	8 (14.0)	
Papillary carcinoma	2 (0.3)		
Gallbladder carcinoma	10 (1.3)		
Other primary malignancy	21 (2.7)		
Mixed HCC/CCC	2 (0.3)	1 (1.8)	
CRLM	95 (12.0)	6 (10.5)	
Neuroendocrine liver metastasis	28 (3.5)	1 (1.8)	
Unspecified liver metastasis	39 (4.9)	2 (3.5)	
Other beningn disorder	51 (6.4)		
FNH	21 (2.7)		
Liver hemangioma	12 (1.5)		
Biliary cyst	13 (1.6)		
Liver adenoma	17 (2.1)	1 (1.8)	
Echinococcus cyst	4 (0.5)	14 (24.6)	

*Note:* Bold font was used when the *p*‐values were < 0.05. This should make it easier to identify significant results.

Abbreviations: ASA = American society of anesthesiologists, avg = average, BMI = body mass index, CCC = cholangiocarcinoma, CRLM = colorectal livermetastasis, DPPHR = duodenum preserved pancreatic head resection, FNH = focal nodular hyperplasia, HCC = hepatocellular Carcinoma, IPMN = intraductal papillary mucinous neoplasm, NET = neuroendocrine tumor, PanIn = pancreatic intraepitheliel neoplasia, PD = pancreaticoduodenectomy, PPPD = pylorus preserved pancreaticoduodenectomy, SD = standard deviation.

**FIGURE 1:**
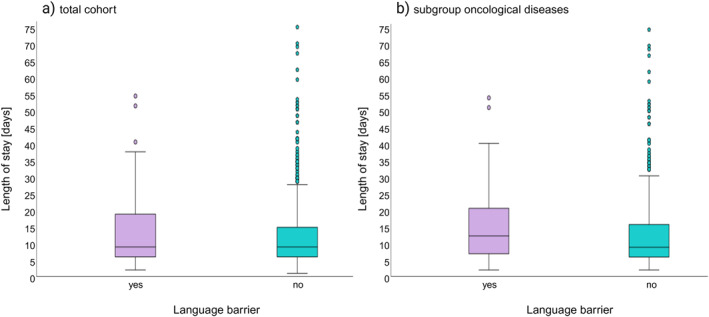
Influence of language barriers on postoperative length of hospital stay (days). (a) Total cohort, (b) Subgroup analysis of patients with oncological diseases. Green: no language barrier; Purple: language barrier present.

Postoperative outcomes are shown in Table 2. We found no significant differences in length of stay between the two groups (12.8 days; (CI 95%: 11.9–13.7) versus 14.4 days; (CI 95%: 11.1–17.7); *p* = 0.320 (Figure 1a). In a multiple linear regression analysis, we additionally examined the association between language barrier and postoperative length of stay while adjusting for potential confounders (age and type of surgical procedure) (Table 3). This model explained approximately 19.7% of the variance in length of stay (adjusted *R* ^2^ = 0.197; SE = 11.1). A language barrier was not independently associated with length of stay (*B* = −1.720; *p* = 0.282; CI 95%:−4.8–1.4) in our model. In contrast major liver surgery (left/right hepatectomies), as well as total pancreatectomy and pancreaticoduodenectomy were significantly associated with longer hospitalization, whereas minor liver resections and Duodenum preserved pancreatic head resections were associated with shorter stays. No evidence of multicollinearity was detected (all VIF < 3). No differences were observed in complication rate or hospital readmissions between the two groups.

**TABLE 2:** Postoperative outcomes (overall study cohort).

Variable	No language barrier (*n* = 791)	Language barrier (*n* = 57)	*p* value
Primary length of stay [avg/SD]	12.8 ± 12.4	14.4 ± 12.1	0.320
Mortality *n* (%)	51 (6.4)	3 (5.3)	0.724
Complications *n* (%)	488 (61.7)	38 (66.7)	0.455
Pneumonia	64 (8.1)	8 (14.0)	0.064
Pleural effusion	93 (11.8)	10 (17.5)	0.099
Respiratory failure	57 (7.2)	3 (5.3)	0.644
Surgical site infection	74 (9.4)	8 (14.0)	0.086
Urinary tract infection	34 (4.3)	3 (5.3)	0.527
Deep venous thrombosis	5 (0.6)	1 (1.8)	0.224
Pulmonary embolus	23 (2.9)	4 (7.0)	**0.026**
Acute kidney injury	90 (11.4)	6 (10.5)	0.947
Paralytic ileus	27 (3.4)	5 (8.8)	**0.047**
Readmission *n* (%)	80 (10.1)	9 (15.8)	0.233
ERAS compliance (%) [avg/SD]	54.6 ± 8.9	53.9 ± 8.6	0.461
Missing	86 (10.9)	1 (1.6)	
Preadmission (%)	65.7 ± 27.7	58.9 ± 31.5	0.153
Preoperative (%)	64.9 ± 11.7	64.5 ± 11.7	0.990
Intraoperative (%)	57.4 ± 17.4	58.7 ± 16.7	0.377
Postoperative (%)	46.2 ± 15.5	46.9 ± 12.9	0.863
Mobilization POD 0 *n* (%)	178 (22.5)	6 (10.5)	**0.009**
Missing	7 (0.9)	1 (1.8)	
Mobilization POD 1 *n* (%)	569 (71.9)	47 (82.5)	0.075
Missing	16 (2.0)		
Mobilization POD 2 *n* (%)	608 (76.9)	47 (82.5)	0.076
Missing	22 (2.8)		
Mobilization POD 3 *n* (%)	599 (75.7)	52 (91.2)	0.079
Missing	25 (3.2)		
ERAS program education *n* (%)	634 (80.2)	39 (68.4)	**0.035**

*Note:* Bold font was used when the *p*‐values were < 0.05. This should make it easier to identify significant results.

Abbreviation: POD = postoperative day.

**TABLE 3:** Multiple linear regression model for length of stay (overall study cohort).

Dependent variable: Length of stay							
Coefficients	*b*	SE	*β*	*t*	*p*	95% CI	
(Constant)	11,410	3819		2987	0.003	3913	18,907
Age	0.033	0.027	0.040	1202	0.230	−0.021	0.087
Language barrier	−1720	1597	−0.035	−1077	0.282	−4854	1414
Major liver resection	0.423	0.151	0.134	2808	0.005	0.127	0.719
Other liver segmentectomy	−0.265	0.165	−0.086	−1604	0.109	−0.589	0.059
Minor liver resection	−0.665	0.183	−0.196	−3636	0.000	−1024	−0.306
Total pancreatectomy	6303	1113	0.226	5662	0.000	4118	8488
PD/PPPD	7290	1695	0.222	4302	0.000	3964	10,617
Distal resection	−0.306	1932	−0.007	−0.159	0.874	−4099	3486
DPPHR	−5484	1971	−0.122	−2783	0.006	−9353	−1616

*Note: N* = 793; *R* ^2^ = 0.201; corr. *R* ^2^ = 0.197; *F*(9,784) = 22.571; *p* = 0.001.

With respect to our ERAS program, compliance was similar, at approximately 54% in both groups and did not differ within different perioperative phases. Patients with language barrier appeared to mobilize significantly less on day of surgery (*p* = 0.009). We also noted that patients with language barrier were significantly less likely to receive preoperative ERAS education (0.035). Upon closer examination of specific postoperative complications, pulmonary embolism and paralytic ileus appeared to occur more frequently in the group with language barriers. Given the limited comparability of the two cohorts, we decided to perform an additional subgroup analysis including only patients with oncological diseases.

Following this adjustment, we obtained two well‐matched cohorts regarding age and final diagnosis (Table 4). In the subgroup we analyzed a cohort of 610 patients with oncological conditions in total. The American Society of Anesthesiologists (ASA) classification, surgical procedure and final diagnosis were similar between the two groups. Regarding comorbidity, this subgroup of patients with language barriers showed a significant higher prevalence of asthma and chronic hepatitis compared to patients without language barriers. The surgical approach used was significantly different between the two groups (*p* = 0.001). The rate of open surgery was higher in patients without language barriers, but the conversion rate was higher in patients with language barriers. One‐third of the patients had pancreatic ductal adenocarcinoma, followed by hepatocellular carcinoma and colorectal liver metastasis. Postoperative outcomes are shown in Table 5. The subgroup analysis revealed findings consistent with the overall study cohort in terms of hospital length of stay, showing no statistically significant differences (16.5 vs. 13.4 days; CI 95%: 11.8–21.2 vs. 12.5–14.4; *p* = 0.182; Figure 1b). In this subgroup restricted to patients with oncologic diseases we used the same model for multiple linear regression as in the overall study cohort and yielded comparable results (Table 6). This analysis also showed no independent association between the presence of a language barrier and length of stay in this multivariate analysis (*B* = −2.357; *p* = 0.196; CI 95%: −5.9–1.2).

**TABLE 4:** Patient characteristics (subgroup analysis).

Variable	No language barrier (*n* = 574)	Language barrier (*n* = 36)	*p*
Anthropometrics			
Female *n* (%)	252 (43.9)	11 (30.6)	0.117
Age [avg/SD]	63.5 ± 13.1	60.4 ± 14.5	0.158
BMI [avg/SD]	25.5 ± 5.4	26.7 ± 6.6	0.432
ASA classification *n* (%)			0.532
I	7 (1.2)		
II	198 (34.5)	12 (33.3)	
III	354 (61.7)	22 (61.1)	
IV	12 (2.1)	1 (2.8)	
Consumption of noxious substances *n* (%)			
Alcohol	110 (19.2)	0 (0)	0.135
Missing	9 (1.6)	1 (2.8)	
Smoking	110 (19.2)	6 (16.7)	0.739
Missing	8 (1.6)	1 (2.8)	
Comorbidities *n* (%)			
Diabetes mellitus	111 (19.3)	10 (27.8)	0.220
Asthma	23 (4.0)	4 (11.1)	**0.045**
COPD	22 (3.8)	0 (0)	0.231
Cardiovascular disease	289 (50.3)	18 (50.4)	0.959
Previous thromboembolic event	55 (9.6)	4 (11.1)	0.766
Chronic kidney disease	26 (4.5)	2 (5.6)	0.777
Steatotic liver disease	25 (4.4)	3 (8.3)	0.270
Chronic hepatitis	43 (7.5)	7 (19.4)	**0.011**
Livercirrhosis	45 (7.8)	3 (8.3)	0.917
Surgical procedure *n* (%)			0.091
PD	63 (11.0)	2 (5.6)	
PPPD	67 (11.7)	3 (8.3)	
DPPHR	1 (0.2)		
Distal resection	53 (9.2)	3 (8.3)	
Total pancreatectomy	46 (8.0)	2 (5.6)	
Other pancreatic surgery	18 (3.1)	2 (5.6)	
Right hemihepatectomy	21 (3.7)	5 (13.9)	
Extended right hemihepatectomy	28 (4.9)	4 (11.1)	
Left hemihepatectomy	16 (2.8)	3 (8.3)	
Extended left hemihepatectomy	14 (2.4)		
Wedge order minor resection	106 (18.5)	7 (19.4)	
Other segmentectomies	112 (19.5)	4 (11.1)	
Other biliary surgery	29 (5.1)		
Surgical approach *n* (%)			**0.001**
Open	424 (73.9)	21 (58.3)	
Laparoscopic	52 (9.1)	6 (16.7)	
Robotic	78 (13.6)	3 (8.3)	
Converted to open	19 (3.3)	6 (16.7)	
Multivisceral resection *n* (%)	125 (21.8)	5 (13.9)	0.262
Final diagnosis *n* (%)			0.421
Pancreatic cancer	210 (36.6)	10 (27.8)	
NET	18 (3.1)	2 (5.6)	
Hilar cholangiocarcinoma	43 (7.5)	4 (11.1)	
Intrahepatic cholangiocarcinoma	37 (6.4)	2 (5.6)	
HCC	69 (12.0)	8 (22.4)	
Papillary carcinoma	2 (0.3)		
Gallbladder carcinoma	10 (1.7)		
Other primary malignancy	21 (3.7)		
Mixed HCC/CCC	2 (0.3)	1 (2.8)	
CRLM	95 (16.6)	6 (16.7)	
Neuroendocrine liver metastasis	28 (4.9)	1 (2.8)	
Unspecified liver metastasis	39 (6.8)	2 (5.6)	

*Note:* Bold font was used when the *p*‐values were < 0.05. This should make it easier to identify significant results.

Abbreviations: ASA = American society of anesthesiologists, avg = average, BMI = body mass index, CCC = cholangiocarcinoma, CRLM = colorectal livermetastasis, DPPHR = duodenum preserved pancreatic head resection, HCC = hepatocellular carcinoma, NET = neuroendocrine tumor, PD = pancreaticoduodenectomy, PPPD = pylorus preserved pancreaticoduodenectomy, SD = standard deviation.

**TABLE 5:** Postoperative outcomes (subgroup analysis).

Variable	No language barrier (*n* = 574)	Language barrier (*n* = 36)	*p* value
Primary length of stay [avg/SD]	13.4 ± 11.4	16.5 ± 13.4	0.182
Mortality *n* (%)	45 (7.8)	2 (5.6)	0.618
Complications *n* (%)	370 (64.5)	26 (72.2)	0.344
Pneumonia	46 (8.0)	7 (19.4)	**0.006**
Pleural effusion	78 (13.6)	6 (16.7)	0.414
Respiratory failure	44 (7.4)	3 (8.3)	0.758
Surgical site infection	61 (10.6)	8 (22.2)	**0.011**
Urinary tract infection	28 (4.9)	3 (8.3)	0.274
Deep venous thrombosis	5 (0.9)	1 (2.8)	0.173
Pulmonary embolus	17 (3.0)	3 (8.3)	**0.028**
Acute kidney injury	81 (14.1)	6 (16.7)	0.526
Paralytic ileus	19 (3.3)	5 (13.9)	**0.002**
Readmission *n* (%)	58 (10.1)	6 (16.7)	0.106
ERAS Compliance (%) [avg/SD]	53.9 ± 9.3	53.2 ± 9.5	0.690
Preadmission (%)	66.1 ± 27.6	58.5 ± 33.9	0.261
Preoperative (%)	65.4 ± 12.0	65.2 ± 13.4	0.851
Intraoperative (%)	56.5 ± 16.9	57.6 ± 17.5	0.434
Postoperative (%)	44.6 ± 15.6	46.1 ± 14.2	0.653
Mobilization POD 0 *n* (%)	105 (18.3)	1 (2.8)	0.136
Missing *n* (%)	4 (0.7)	1 (2.8)	
Mobilization POD 1 *n* (%)	404 (70.4)	29 (80.6)	0.315
Missing	12 (2.1)		
Mobilization POD 2 *n* (%)	449 (78.2)	29 (80.6)	0.339
Missing	14 (2.4)		
Mobilization POD 3 *n* (%)	444 (77.4)	31 (86.1)	0.310
Missing	18 (3.1)		
ERAS program education *n* (%)	459 (80.0)	24 (66.7)	0.057

*Note:* Bold font was used when the *p*‐values were < 0.05. This should make it easier to identify significant results.

Abbreviation: POD = postoperative day.

**TABLE 6:** Multiple linear regression model for lenght of stay (subgroup analysis oncological diseases).

Dependent variable: Length of stay							
Coefficients	*B*	SE	*β*	*t*	*p*	95% CI	
(Constant)	9777	10,999		0.889	0.374	−11,828	31,381
Age	−0.001	0.033	−0.001	−0.029	0.977	−0.065	0.063
Language barrier	−2357	1822	−0.049	−1293	0.196	−5936	1223
Major liver resection	0.451	0.160	0.163	2818	0.005	0.137	0.766
Other liver segmentectomy	−0.318	0.181	−0.110	−1753	0.080	−0.674	0.038
Minor liver resection	−0.712	0.202	−0.220	−3527	< 0.001	−1108	−0.315
Total pancreatectomy	6083	1138	0.260	5345	< 0.001	3847	8318
PD/PPPD	6119	1797	0.218	3405	< 0.001	2589	9649
Distal resection	−0.868	2063	−0.022	−0.421	0.674	−4920	3184
DPPHR	0.578	10,311	0.002	0.056	0.955	−19,676	20,832

*Note: N* = 562; *R* ^2^ = 0.238; adj. *R* ^2^ = 0.226; *F*(9,553) = 19.187; *p* = 0.001.

With respect to our ERAS program, compliance was similar, at approximately 53% in both groups (*p* = 0.690). We did not identify any differences in ERAS compliance across the different perioperative phases, and in contrast to the overall cohort, mobilization rates during the first postoperative days did not differ between the groups. While we observed a trend toward less ERAS education in the language barrier group than in the nonlanguage barrier group, this difference did not reach statistical significance (66.7% vs. 80.0%; CI 95%: 0.66–1.05; *p* = 0.057). Additionally, although not statistically significant, the readmission rate was slightly higher in the language barrier group (16.7% vs. 10.1%; CI 95%: 0.67–1.10; *p* = 0.106). Overall complications and mortality did not differ significantly between the groups, even though we observed approximately 10% more complications in the language barrier group (64.5% vs. 72.2%; CI 95%: 0.96–1.41; *p* = 0.344). In terms of specific complications, we observed that patients with language barriers had higher rates of pneumonia, wound infection, pulmonary embolism and paralytic ileus in this subgroup. The rate of pneumonia was more than twice that in the language barrier group compared with the nonlanguage barrier group (19.4% vs. 8.0%; CI 95%: 1.04–3.72; *p* = 0.006). Wound infections were more common in patients with language barriers than in patients without language barriers (22.2% vs. 10.6%; CI 95%: 1.11–3.59; *p* = 0.011). The incidence of pulmonary embolism in the language barrier group was almost three times greater than that in the nonlanguage barrier group (8.3% vs. 3.0%; CI 95%: 1.36–9.02; *p* = 0.0028). We also observed a significant difference in the occurrence of paralytic ileus, which was more than four times greater in the language barrier group than in the nonlanguage barrier group (13.9% vs. 3.3%; CI 95%: 1.24–6.98; *p* = 0.002).

## 4 Discussion

This study set out to investigate the potential impact of language barriers on perioperative outcomes in patients undergoing hepatobiliary and pancreatic surgery. To our knowledge, this is the first study investigating outcomes from patients with limited language proficiency receiving surgical care in Germany. According to our data, patients with language barriers remain a small population; approximately 6% of our patients had a language barrier. In this study we investigated differences in length of hospital stay, ERAS‐compliance, mobilization habits, preoperative education frequency, postoperative complications and readmission rates between the two groups. No significant differences were observed regarding length of stay and patients were comparable in terms of comorbidities. However, comparability between the cohorts was limited as patients in the language barrier group were, on average, 10 years younger. Patients with a language barrier were less likely to receive preoperative ERAS education from an ERAS Nurse and mobilized significantly less on the day of surgery. The reasons for the lower rate of preoperative ERAS education among patients with language barriers cannot be determined within the scope of this study. However, this could be primarily related to organizational factors, including the limited availability of interpreters, the additional time required for consultations with interpreting services, and, in some cases, short‐term surgery planning, which may have further limited the feasibility of preoperative ERAS education for patients with language barriers.

Statistically significant differences were noted for certain complications; however, these findings should be interpreted with caution due to the rarity of the events and the limited cohort size and they warrant further validation in lager studies. To enhance comparability, we performed a subgroup analysis with patients with oncological diseases, which yielded results consistent with those observed in the overall cohort.

We saw an enormous variety of 19 different spoken languages in our study population; this underscores the complexity of communication in this specific group and highlights the need for structured multilingual support within clinical pathways. In line with already published studies, our data show that professional interpreting services are rarely used in hospitals [17, 21]. While an interpreter is required to be present for preoperative preparation in order to obtain consent for the operation, it is unclear how postoperative information about the surgical procedure and diagnosis are delivered to the patient. Bilingual health care professionals and family members may play an important role within this process. Over a third of the patients with language barriers only had preoperative contact with an interpreter, which could have potentially resulted in incomplete information transfer and may have impaired their understanding of the surgical procedure, disease and recommended beneficial behaviors. In line with previous studies from other fields, our data indicate that patients with language barriers might be at a disadvantage in their postoperative recovery process [27, 31]. After adjusting for surgical procedure in a multiple linear regression model in the overall cohort, as well as in our subgroup analysis the presence of a language barrier did not independently influence the length of stay in hospital. In contrast to our initial expectations, we observed similar ERAS compliance in the two groups. A potential explanation for this might lie in ERAS compliance itself, which includes several elements that are dependent on the actions of the health care professionals rather than on patient engagement. In our study, patients with language barriers had significantly higher rates of specific postoperative complications, such as paralytic ileus and pulmonary embolism. These findings could indicate that this patient population may be particularly susceptible to developing these kinds of complications. Although casual relationships cannot be established in the nature of our study, it is notable that prevention of these kinds of complications relies heavily on factors such as successful mobilization and compliance with prescribed medication regimens [43, 44]. As these preventive measures require patients to be well informed and actively participate, language barriers may negatively influence their successful establishment.

A recent systematic review by Lent et al. investigated the effectiveness of strategies for mitigating language barriers [45]. The authors concluded that a health care provider sharing the same language and professional interpreters were the most effective strategies for overcoming language barriers [45]. If relatives are consulted as translators, this should be done with care and caution. This option poses significant risks in terms of translation accuracy and can lead to ethical challenges for health care institutions [46]. Relatives have special relationships with patients. Depending on how the relationship is structured, it can lead to a distortion of information and, under certain circumstances, to bias that can impair the overall treatment of the patient. Relatives should also be protected from bearing the burden of communicating crucial information such as prognoses and diagnoses to a close family member. However, a deeper and better understanding is probably needed not only on a linguistic level but also on socioeconomic and cultural levels in order to address the challenges in clinical care.

This retrospective study has several limitations. It was a single center study, and we were only able to analyze a small group of patients with language barriers. As shown in previous studies, language barriers in clinical practice are not assessed regularly and are mostly subjectively judged by health care professionals within the first consultation [21]. This has the potential of underestimating the difficulties nonnative speakers might have with understanding complex health‐related information, including the fact that some patients might not be identified as patients with a language barrier [21]. This also applies to our study. It is possible that in some cases where the surgeons consulted did not consider the language barrier to be significant, no interpreting services were used, even though a language barrier existed. This may have resulted in patients with language barrier being included in the group classified as having no language barrier.

Our findings provide important initial insights into how communication barriers due to differences in language proficiency can affect clinical outcomes in this complex surgical population. Currently, there is a remarkable lack of research in Germany addressing specific risks of patients with language barriers in surgical settings. Our results suggest that this cohort may face disadvantages in postoperative recovery and could potentially be at risk for certain complications. This underscores the need for ongoing research especially with large patient cohorts to detect more rare time events such as pulmonary embolisms and conduct more precise statistical analysis. Moreover, it may be of particular interest to explore how artificial intelligence could help to improve communication between healthcare professionals and patients with language barriers. Equally important are qualitative studies focusing on the experiences and perceptions of patient with language barriers in surgical settings, as they may provide valuable insights into how to improve patient‐centered care and successful ERAS implementation in such cohorts. From a clinical perspective, the identification of a language barrier in particular appears to be a critical moment in the care process. Standardized and multilingual screening tools could support the establishment of effective and comprehensible communication thereby fostering patient autonomy, shared decision‐making and patient participation. The use of professional interpreters is critical for safe and patient‐centered care from admission and through the patient's stay to discharge.

## Author Contributions

**Freya Brodersen:** conceptualization, data curation, formal analysis, investigation, methodology, project administration, writing – original draft. **Jana Hinz:** conceptualization, data curation, investigation, writing – review and editing. **Sinja Friedl:** conceptualization, supervision. **Faik Güntac Uzunoglu:** supervision. **Asmus Heumann:** supervision. **Tarik Ghadban:** supervision, writing – review and editing. **Ramez Wahib:** methodology, supervision. **Thilo Welsch:** methodology, supervision, writing – review and editing. **Thilo Hackert:** resources, supervision. **Sidra Khan‐Gökkaya:** conceptualization, methodology, project administration, validation, supervision, writing – review and editing.

## Funding

The authors have nothing to report.

## Conflicts of Interest

The authors declare no conflicts of interest.

## Data Availability

The data that support the findings of this study are available from the corresponding author upon reasonable request.

## References

[1] J. K. O'Toole , W. Alvarado‐Little , and C. J. W. Ledford , “Communication With Diverse Patients: Addressing Culture and Language,” Pediatric Clinics of North America 66, no. 4 (2019): 791–804, 10.1016/j.pcl.2019.03.006. 31230623

[2] L. S. Karliner , E. A. Jacobs , A. H. Chen , and S. Mutha , “Do Professional Interpreters Improve Clinical Care for Patients With Limited English Proficiency? A Systematic Review of the Literature,” Health Services Research 42, no. 2 (2007): 727–754, 10.1111/j.1475-6773.2006.00629.x. 17362215 PMC1955368

[3] D. de Moissac and S. Bowen , “Impact of Language Barriers on Quality of Care and Patient Safety for Official Language Minority Francophones in Canada,” Journal of Patient Experience 6, no. 1 (2019): 24–32, 10.1177/2374373518769008. 31236448 PMC6572938

[4] A. K. Gupta , O. Kleinig , S. Tan , et al., “Lost in Translation: The Impact of Language Barriers on the Outcomes of Patients Receiving Coronary Artery Revascularization,” Cardiovascular Revascularization Medicine 52 (2023): 94–98, 10.1016/j.carrev.2023.03.016. 36990850

[5] L. Diamond , K. Izquierdo , D. Canfield , K. Matsoukas , and F. Gany , “A Systematic Review of the Impact of Patient‐Physician Non‐English Language Concordance on Quality of Care and Outcomes,” Journal of General Internal Medicine 34, no. 8 (2019): 1591–1606, 10.1007/s11606-019-04847-5. 31147980 PMC6667611

[6] A. Fernandez , D. Schillinger , E. M. Warton , et al., “Language Barriers, Physician‐Patient Language Concordance, and Glycemic Control Among Insured Latinos With Diabetes: The Diabetes Study of Northern California (DISTANCE),” Journal of General Internal Medicine 26, no. 2 (2011): 170–176, 10.1007/s11606-010-1507-6. 20878497 PMC3019330

[7] A. John‐Baptiste , G. Naglie , G. Tomlinson , et al., “The Effect of English Language Proficiency on Length of Stay and In‐Hospital Mortality,” Journal of General Internal Medicine 19, no. 3 (2004): 221–228, 10.1111/j.1525-1497.2004.21205.x. 15009776 PMC1492154

[8] A. Khan , H. S. Yin , C. Brach , et al., “Association Between Parent Comfort With English and Adverse Events Among Hospitalized Children,” JAMA Pediatrics 174, no. 12 (2020): e203215, 10.1001/jamapediatrics.2020.3215. 33074313 PMC7573792

[9] A. Chauhan , M. Walton , E. Manias , et al., “The Safety of Health Care for Ethnic Minority Patients: A Systematic Review,” International Journal for Equity in Health 19, no. 1 (2020): 118, 10.1186/s12939-020-01223-2. 32641040 PMC7346414

[10] S. A. Fox and J. A. Stein , “The Effect of Physician‐Patient Communication on Mammography Utilization by Different Ethnic Groups,” Medical Care 29, no. 11 (1991): 1065–1082, 10.1097/00005650-199111000-00001. 1943268

[11] M. Pandey , R. G. Maina , J. Amoyaw , et al., “Impacts of English Language Proficiency on Healthcare Access, Use, and Outcomes Among Immigrants: A Qualitative Study,” BMC Health Services Research 21, no. 1 (2021): 741, 10.1186/s12913-021-06750-4. 34311712 PMC8314461

[12] K. Fiscella , P. Franks , M. P. Doescher , and B. G. Saver , “Disparities in Health Care by Race, Ethnicity, and Language Among the Insured: Findings From a National Sample,” Medical Care 40, no. 1 (2002): 52–59, 10.1097/00005650-200201000-00007. 11748426

[13] A. Lebano , S. Hamed , H. Bradby , et al., “‘Migrants’ and Refugees’ Health Status and Healthcare in Europe: A Scoping Literature Review,” BMC Public Health 20, no. 1 (2020): 1039, 10.1186/s12889-020-08749-8. 32605605 PMC7329528

[14] R. Calvillo‐Ortiz , J. C. Polanco‐Santana , M. Castillo‐Angeles , et al., “Language Proficiency and Survival in Pancreatic Cancer: A Propensity Score‐Matched Analysis,” Journal of Gastrointestinal Surgery 26, no. 1 (2022): 94–103, 10.1007/s11605-021-05081-3. 34258672

[15] S. Duraiswamy , S. J. Rubin , Y. Kim , T. Mur , and H. A. Edwards , “Limited English Proficiency and Head and Neck Cancer Outcomes,” American Journal of Otolaryngology 43, no. 3 (2022): 103470, 10.1016/j.amjoto.2022.103470. 35427938

[16] A. Bischoff and K. Denhaerynck , “What Do Language Barriers Cost? an Exploratory Study Among Asylum Seekers in Switzerland,” BMC Health Services Research 10, no. 1 (2010): 248, 10.1186/1472-6963-10-248. 20731818 PMC2939598

[17] L. Gerchow , L. R. Burka , S. Miner , and A. Squires , “Language Barriers Between Nurses and Patients: A Scoping Review,” Patient Education and Counseling 104, no. 3 (2021): 534–553, 10.1016/j.pec.2020.09.017. 32994104 PMC8011998

[18] S. P. Manuel , Z. K. Chia , K. P. Raygor , and A. Fernández , “Association of Language Barriers With Process Outcomes After Craniotomy for Brain Tumor,” Neurosurgery 91, no. 4 (2022): 590–595, 10.1227/neu.0000000000002080. 35857019 PMC10552977

[19] S. P. Manuel , K. Nguyen , L. S. Karliner , D. T. Ward , and A. Fernandez , “Association of English Language Proficiency With Hospitalization Cost, Length of Stay, Disposition Location, and Readmission Following Total Joint Arthroplasty,” JAMA Network Open 5, no. 3 (2022): e221842, 10.1001/jamanetworkopen.2022.1842. 35267037 PMC8914571

[20] M. Roy , N. Purington , M. Liu , D. W. Blayney , A. W. Kurian , and L. Schapira , “Limited English Proficiency and Disparities in Health Care Engagement Among Patients With Breast Cancer,” JCO Oncology Practice 17, no. 12 (2021): e1837–e1845, 10.1200/op.20.01093. 33844591 PMC9810131

[21] F. van Rosse , M. de Bruijne , J. Suurmond , M.‐L. Essink‐Bot , and C. Wagner , “Language Barriers and Patient Safety Risks in Hospital Care. A Mixed Methods Study,” International Journal of Nursing Studies 54 (2016): 45–53, 10.1016/j.ijnurstu.2015.03.012. 25840899

[22] Q. Ngo‐Metzger , D. H. Sorkin , R. S. Phillips , et al., “Providing High‐Quality Care for Limited English Proficient Patients: The Importance of Language Concordance and Interpreter Use,” supplement, Journal of General Internal Medicine 22, no. S2 (2007): 324–330, 10.1007/s11606-007-0340-z. 17957419 PMC2078537

[23] H. Joo , A. Fernández , E. C. Wick , G. Moreno Lepe , and S. P. Manuel , “Association of Language Barriers With Perioperative and Surgical Outcomes: A Systematic Review,” JAMA Network Open 6, no. 7 (2023): e2322743, 10.1001/jamanetworkopen.2023.22743. 37432686 PMC10336626

[24] T. Feeney , M. Cassidy , Y. Tripodis , et al., “Association of Primary Language With Outcomes After Operations Typically Performed to Treat Cancer: Analysis of a Statewide Database,” Annals of Surgical Oncology 26, no. 9 (2019): 2684–2693, 10.1245/s10434-019-07484-8. 31187361

[25] X. Dai , M. A. Ryan , A. C. Clements , et al., “The Effect of Language Barriers at Discharge on Pediatric Adenotonsillectomy Outcomes and Healthcare Contact,” Annals of Otology, Rhinology & Laryngology 130, no. 7 (2021): 833–839, 10.1177/0003489420980176. 33319598

[26] L. S. Karliner , S. E. Kim , D. O. Meltzer , and A. D. Auerbach , “Influence of Language Barriers on Outcomes of Hospital Care for General Medicine Inpatients,” Journal of Hospital Medicine 5 (2010): 276–282, 10.1002/jhm.658. 20533573

[27] E. W. Tang , J. Go , A. Kwok , et al., “The Relationship Between Language Proficiency and Surgical Length of Stay Following Cardiac Bypass Surgery,” European Journal of Cardiovascular Nursing 15, no. 6 (2016): 438–446, 10.1177/1474515115596645. 26198643

[28] M. R. H. Castro , H. Schwartz , S. Hernandez , et al., “The Association of Limited English Proficiency With Morbidity and Mortality After Trauma,” Journal of Surgical Research 280 (2022): 326–332, 10.1016/j.jss.2022.07.044. 36030609

[29] C. A. Plancarte , P. Hametz , and W. N. Southern , “Association Between English Proficiency and Timing of Analgesia Administration After Surgery,” Hospital Pediatrics 11 (2021): 1199–1204, 10.1542/hpeds.2020-005766. 34654728

[30] A. L. Hines , R. M. Andrews , E. Moy , M. Barrett , and R. Coffey , “Disparities in Rates of Inpatient Mortality and Adverse Events: Race/Ethnicity and Language as Independent Contributors,” International Journal of Environmental Research and Public Health 11, no. 12 (2014): 13017–13034, 10.3390/ijerph111213017. 25514153 PMC4276659

[31] N. Chicoine , S. Greenberg , D. Barry , A. Dick , and H. Cockrell , “Association Between Language, Interpreter Use, and Pediatric Surgical Outcomes,” Journal of Pediatric Surgery 60, no. 3 (2025): 162104, 10.1016/j.jpedsurg.2024.162104. 39808857

[32] O. Strobel , J. Neoptolemos , D. Jäger , and M. W. Büchler , “Optimizing the Outcomes of Pancreatic Cancer Surgery,” Nature Reviews Clinical Oncology 16, no. 1 (2019): 11–26, 10.1038/s41571-018-0112-1. 30341417

[33] C. Krautz , C. Gall , O. Gefeller , et al., “In‐hospital Mortality and Failure to Rescue Following Hepatobiliary Surgery in Germany—A Nationwide Analysis,” BMC Surgery 20, no. 1 (2020): 171, 10.1186/s12893-020-00817-5. 32727457 PMC7388497

[34] P. J. Kneuertz , H. A. Pitt , K. Y. Bilimoria , et al., “Risk of Morbidity and Mortality Following Hepato‐Pancreato‐Biliary Surgery,” Journal of Gastrointestinal Surgery 16, no. 9 (2012): 1727–1735, 10.1007/s11605-012-1938-y. 22760965

[35] S. Kawakatsu , J. Yamaguchi , T. Mizuno , et al., “Early Prediction of a Serious Postoperative Course in Perihilar Cholangiocarcinoma: Trajectory Analysis of the Comprehensive Complication Index,” Annals of Surgery 277, no. 3 (2023): 475–483, 10.1097/sla.0000000000005162. 34387204

[36] C. Kuemmerli , C. Tschuor , M. Kasai , et al., “Impact of Enhanced Recovery Protocols After Pancreatoduodenectomy: Meta‐Analysis,” British Journal of Surgery 109, no. 3 (2022): 256–266, 10.1093/bjs/znab436. 35037019

[37] L. Noba , S. Rodgers , C. Chandler , A. Balfour , D. Hariharan , and V. S. Yip , “Enhanced Recovery After Surgery (ERAS) Reduces Hospital Costs and Improve Clinical Outcomes in Liver Surgery: A Systematic Review and Meta‐Analysis,” Journal of Gastrointestinal Surgery 24, no. 4 (2020): 918–932, 10.1007/s11605-019-04499-0. 31900738 PMC7165160

[38] O. Ljungqvist , “Enhanced Recovery After Surgery: A Paradigm Shift in Perioperative Care,” in Enhanced Recovery After Surgery: A Complete Guide to Optimizing Outcomes 2020, 3‐9.

[39] E. von Elm , D. G. Altman , M. Egger , S. J. Pocock , P. C. Gøtzsche , and J. P. Vandenbroucke , “The Strengthening the Reporting of Observational Studies in Epidemiology (STROBE) Statement: Guidelines for Reporting Observational Studies,” PLoS Medicine 4, no. 10 (2007): e296, 10.1371/journal.pmed.0040296. 17941714 PMC2020495

[40] O. Ljungqvist and M. Hübner , “Introducing Enhanced Recovery Programs Into Practice: Lessons Learned From the ERAS Society Implementation Program,” SAGES/ERAS Society Manual of Enhanced Recovery Programs for Gastrointestinal Surgery (2015): 215–226.

[41] O. Beetz , A. Sarisin , A. Kaltenborn , J. Klempnauer , M. Winkler , and G. Grannas , “Multivisceral Resection for Adenocarcinoma of the Pancreatic Body and Tail‐a Retrospective Single‐Center Analysis,” World Journal of Surgical Oncology 18, no. 1 (2020): 218, 10.1186/s12957-020-01973-x. 32819373 PMC7441692

[42] W. Hartwig , C. M. Vollmer , A. Fingerhut , et al., “Extended Pancreatectomy in Pancreatic Ductal Adenocarcinoma: Definition and Consensus of the International Study Group for Pancreatic Surgery (ISGPS),” Surgery 156 (2014): 1–14, 10.1016/j.surg.2014.02.009. 24856668

[43] A. Turan , A. K. Khanna , J. Brooker , et al., “Association Between Mobilization and Composite Postoperative Complications Following Major Elective Surgery,” JAMA Surgery 158, no. 8 (2023): 825–830, 10.1001/jamasurg.2023.1122. 37256591 PMC10233451

[44] S. M. Fernando , A. Tran , W. Cheng , et al., “VTE Prophylaxis in Critically Ill Adults: A Systematic Review and Network Meta‐Analysis,” Chest 161, no. 2 (2022): 418–428, 10.1016/j.chest.2021.08.050. 34419428

[45] L. G. G. van Lent , N. G. Yilmaz , S. Goosen , et al., “Effectiveness of Interpreters and Other Strategies for Mitigating Language Barriers: A Systematic Review,” Patient Education and Counseling 136 (2025): 108767, 10.1016/j.pec.2025.108767. 40179546

[46] A. Squires , “Strategies for Overcoming Language Barriers in Healthcare,” Nursing Management 49, no. 4 (2018): 20–27, 10.1097/01.numa.0000531166.24481.15. PMC8697718 29528894

